# Social determinants of health and sex differences: insights from NIH Specialized Centers of Research Excellence (SCORE)

**DOI:** 10.1186/s13293-026-00936-3

**Published:** 2026-06-08

**Authors:** Sherry A. McKee, Lin Chang, Izzuddin M. Aris

**Affiliations:** 1 Department of Psychiatry, Yale School of Medicine, New Haven, CT USA; 2 Department of Medicine, David Geffen School of Medicine, University of California, Los Angeles, CA USA; 3 Department of Population Medicine, Harvard Medical School and Harvard Pilgrim Health Care Institute, Boston, MA USA

Nearly two decades ago, the World Health Organization established its commission on the social determinants of health (SDOH) to galvanize global action around the social factors that profoundly shape the health of individuals, families, communities, and populations [1]. These determinants are typically grouped into five broad domains: health care access and quality; economic stability; neighborhood and built environment; social and community context; and education access and quality [2]. Critically, SDOH influence access to health-promoting resources, such as nutritious food, transportation, safe housing, and reliable utilities, as well as economic opportunities that serve as foundational drivers of optimal health. In contrast, certain populations may experience limited access to the same resources, services, and opportunities due to language, socioeconomic, or geographic barriers, contributing to poor health. A robust and growing body of evidence underscores the central role of SDOH as key drivers of population-level health disparities. Where we are born, grow, live, work and age have been shown to differentially impact life expectancy between high- and low-income countries by 33 years [3]. Addressing SDOH is fundamental for improving health.

Importantly, women’s health is particularly sensitive to these social and structural influences. Factors such as income, education, employment, geographic location, family dynamics, and broader policy environments all intersect to shape women’s health experiences and outcomes [4]. Social forces that give rise to differences in men’s and women’s lives can interact with biological differences in complex ways, often compounding or masking the latter. These determinants, through a range of pathways, not only influence health behaviors and access to healthcare but also impact the onset, presentation, and progression of various diseases. Moreover, women may encounter systemic challenges within the health care system itself, including bias in clinical practice and patient-centered care, which can further affect health outcomes [5]. This special collection of articles in Biology of Sex Differences explores the pivotal role of SDOH in driving not only disparities in women’s health but also the sex-based differences in disease risk, manifestation, and outcomes. Understanding these interwoven factors is essential for advancing both women’s health and broader efforts to improve health across populations. The collection of articles features studies from the NIH-funded Specialized Centers of Research Excellence (SCORE) investigators.

The SCORE program for the study of sex differences is a flagship program of the Office of Research on Women’s Health (ORWH) that was started as a P50 initiative in 2002, then called Specialized Centers of Research (SCOR). In 2018, the SCOR program was expanded to a U54 cooperative agreement, SCORE, by adding a new feature, Career Enhancement Core (CEC), to train the next generation of scientists for women’s health research.

Since the inception of the program in 2002, SCORE is the only NIH’s center-level disease-agnostic program focused on sex differences. The program aims to translate scientific knowledge about how diseases affect women and men differently into new treatments that improve clinical care. It is worth mentioning that the P50-SCOR program preceded the now-landmark NIH Sex as a Biological Variable (SABV) policy (NOT-OD-15-102) by more than a decade. The SCORE program’s emphasis on the consideration of sex as a biological variable (SABV) is integral to scientific rigor, reproducibility, and transparency.

The overall objectives of the SCORE Centers of Excellence is to promote disease-agnostic, investigator initiated, interdisciplinary, and translational research by functioning independently and collaboratively to (1) expedite the development and application of new knowledge on sex differences; (2) lead as a regional/national Center of Research Excellence on sex differences; (3) serve as a potential resource and repository for data, biospecimens, and sex differences research tools; (4) educate the broader scientific community; (5) train the next generation of researchers; and (6) disseminate information on sex differences as it influences the etiology, trajectory, and treatment/prevention of human disease with special emphasis to the health of women.

This collection of articles in *Biology of Sex Differences* underscores how SDOH shape women’s health across the life course and intersect with biological differences to influence disease risk, presentation, and outcomes (see Table 1). From rural cardiovascular health disparities in Georgia to hypertensive disorders of pregnancy that reverberate into midlife sleep disturbance, these studies reveal the profound and lasting consequences of structural, environmental, and socioeconomic factors on women’s health trajectories. Environmental exposures such as neighborhood disadvantage and lack of greenspace compound biological vulnerability, while psychosocial challenges, such as stress, trauma, and socioeconomic strain, emerge as consistent drivers of risk for cardiovascular disease, irritable bowel syndrome (IBS), Takotsubo syndrome, and adverse perinatal outcomes. Together, these studies highlight that environmental factors are as important to health outcomes as genetic or hormonal factors.

**Table 1:** Summary of Research Findings on Social Determinants of Health at Specialized Centers of Research Excellence (SCORE) on Sex Differences

Citation	Study description	Social Determinants of Health as Predictors of Health Outcomes				
		Healthcare access and quality	Education access and quality	Social and community context	Economic stability	Neighborhood and built environment
Holcomb et al., (2025) [6]	SODH in maternal mental health screening and treatment engagement during the perinatal period. (F only sample)	NA	Education was NS	Disabilities and mental health concerns were unmet needs in pregnant women. Family and community support was NS.	Financial strain and food insecurity were unmet needs in pregnant women	NA
Vernon et al., (2025) [7]	Mixed quantitative and qualitative study to examine cardiovascular health (CVH) risk factors among women in rural Georgia, to identify SODH as barriers to care. (F only sample)	Affordability and limited access to care were barriers to treatment for CVH.	Lack of access to health education identified as barrier to CVH care.	NA	Financial strain identified as a barrier to CVB care.	Women living in rural vs. urban areas had greater CVH risk. Women identified lack of transportation and lack of access to the internet as a barrier to CVH care.
Heydari et al., (2025) [8]	Longitudinal study (Project Viva) testing whether having high blood pressure around pregnancy was linked to worse sleep in middle age. (F only sample)	NA	Women who were not college graduates and who experienced high blood pressure during pregnancy had worse sleep outcomes in midlife.	NA	Women with household income >$70,000 and who experienced high blood pressure during pregnancy had worse sleep outcomes.	NA
Rifas-Shiman et al., (2025) [9]	Longitudinal cohort study examining how street-view level greenspace is associated with cardiovascular health in midlife women. (F only sample)	NA	NA	NA	NA	Greater street-view greenspace exposure, particularly trees, was associated with higher overall cardiovascular health scores ~ 5 years later, including healthier behaviors and biomedical measures.
Obrutu et al., (2025) [10]	Case-control study evaluating the relationship between psychosocial characteristics and Takutsubo. Syndrome (TTS)	NA	NA	Compared to participants confirmed to be non-TTS, those with adjudication-confirmed TTS had worse mean psychosocial scores for post-traumatic stress disorder and depression.	NA	NA
Arnold et al., (2025) [11]	Cross-sectional study evaluating childhood and adult trauma, and HIV status on mental health and inflammation in women. (F only sample)	NA	NA	Women with higher levels of childhood maltreatment show greater PTSD and depression symptoms, and higher inflammation.	High socioeconomic disadvantage (low income, low education, disability support, history of arrest) was observed in women with and without HIV likely contributed to lack of major differences in mental health and inflammation outcomes.	NA
Kling et al., (2025) [12]	Cross-sectional study evaluating the impact of SDOH on the likelihood of hormone therapy (HT) utilization among midlife women. (F only sample)	NA	Lower (vs. higher) education level was associated with lower odds of HT utilization	Women who were unpartnered (vs. those who were married) were less likely to utilize HT	NA	NA
Kilpatrick et al., (2025) [13]	A cross-sectional study examining how neighborhood disadvantage is associated with brain structure, gut microbiome, and clinical data in irritable bowel syndrome (IBS). (F vs. M sample)	NA	NA	NA	NA	Area deprivation index (ADI), F + M, but ADI associated with changes in cortical morphology and microbiome composition in IBS-F but not IBS-M
Holcomb et al., (2025) [14]	Cross-sectional study on prevalence of sex differences in SDOH in primary care setting. (F vs. M sample)	NA	Health literacy, M > F	Intimate partner violence, depression, stress, social connections F > M; Alcohol and tobacco use, M > F	Financial strain, food insecurity, and housing instability: all F > M	Transportation barriers, F > M
Binod et al., (2025) [15]	Cross-sectional study on how adverse childhood experiences (ACE) influences brain function, gut microbiome, and symptom severity in women with irritable bowel syndrome (IBS). (F only sample)	NA	NA	High ACE in IBS-F associated with altered brain connectivity and microbiome changes vs. healthy F	NA	NA
Peltier et al., (2025) [16]	Epidemiological study of alcohol consumption during pregnancy. (F only sample)	NA	NA	Criminal justice involvement and psychiatric distress predicted alcohol consumption during pregnancy	Not requiring full- time employment predicted alcohol consumption during pregnancy	NA
Chavez et al., (2025) [17]	Epidemiological study of SODH predictors of substance use treatment outcomes in publicly funded treatment. (F vs. M sample)	Results were similar for women and men for healthcare access (F = M)	Educational attainment did not impact on treatment outcomes for either sex (NS)	NA	Lack of employment and housing was a larger predictor for treatment failure for women vs. men (F > M)	NA

NA: Not assessed; NS: Not significant; F = M: Effect was significant and equal in females and males; F > M: Effect was significantly greater in females vs. males; F < M: Effect was significantly reduced in females vs. males

A recurring theme across these investigations is the intersection of SDOH with sex-specific vulnerabilities in disease pathways. Several studies demonstrate how pregnancy, menopause, and midlife transitions amplify the impact of social adversity, whether through alcohol use during pregnancy, variable access to hormone therapy for menopausal symptoms, or the compounding effects of adverse childhood experiences on brain-gut-microbiome interactions in women with IBS. Others reveal how psychosocial stressors such as financial insecurity, intimate partner violence, depression, and post-traumatic stress disorder disproportionately affect women’s disease burden and clinical outcomes. Importantly, the role of systemic barriers, limited access to providers in rural areas, biases in screening and treatment engagement for maternal mental health, and differential patterns of substance use disorder treatment completion, underscores the urgent need for structural interventions that extend beyond direct patient care.

Equally important are new methodological approaches and conceptual tools to better capture the complexity of sex differences in biomedical research. Methodological innovations, alongside large-scale mixed-methods and multi-omics analyses, advance our ability to link SDOH with biological mechanisms, from immune activation and brain morphology to cardiovascular risk factors and gut microbial signatures. Collectively, these papers make clear that progress in women’s health requires integrating the study of SDOH with sex differences research.

By fostering interdisciplinary, translational research, SCORE Centers have accelerated discoveries that bridge biological, behavioral, and social domains. Notably, the program’s emphasis on SABV continues to set the standard for scientific rigor, reproducibility, and relevance. The contributions in this special issue demonstrate how integrating social and structural determinants with biological sex differences yields actionable insights that can transform women’s health research and care.
